# Editorial: The role of pharmacogenomics in addressing health disparities: the path, the promise, and the barriers

**DOI:** 10.3389/fgene.2023.1233045

**Published:** 2023-06-08

**Authors:** Youssef M. Roman

**Affiliations:** Pharmacotherapy and Outcomes Science Department, School of Pharmacy, Virginia Commonwealth University, Richmond, VA, United States

**Keywords:** diversity & inclusion, race and ethnicities, health equity, health disparities, minorities, genomics, implementation, pharmacogenomics

## Introduction

Despite rapid advancements in genetic technologies to detect disease and optimize treatment, utilizing genomic information to ameliorate health disparities in underserved populations remains a great need for research and implementation (Roberts et al., 2019). High variability in treatment response may significantly contribute to the health disparities observed in specific subpopulations leading to disproportionate disease burden (Fine et al., 2005). While genetics, among other risk factors, can affect disease liability among some population groups, strategies to use genetic information to inform optimal disease management—maximizing benefit and minimizing risk—also vary by population (West et al., 2017). Substantive efforts have been directed to demonstrate the utility of pharmacogenomics (PGx) in tailoring therapies, yet there is a paucity of research on the role of PGx in addressing health disparities (Martin et al., 2017). Pharmacogenomics is a robust theoretical framework to improve healthcare delivery; however, this tool has not been adopted as an operational framework to reduce health disparities. Therefore, this Research Topic was commissioned to provide a platform to share real-world barriers and facilitators to garner the benefits of pharmacogenomics.

## Pharmacogenomics in the post-pandemic era

The COVID-19 pandemic not only tested the resiliency of our healthcare system but may have also highlighted critical measures of preparedness, including preemptive PGx testing, to address future health crises. The availability of the patient’s PGx profile in acute settings could significantly change the course of therapy and optimize patient care. While reactive PGx testing is the predominant approach for delivering patient care, the COVID-19 pandemic underscored the potential utility of preemptive PGx testing as Al-Mahayri described. Given that the pandemic disproportionately impacted individuals with multiple comorbidities who presumably have the highest medication burden, patients’ PGx information readiness during acute illnesses may optimize drug selection or avert potential drug-drug-gene interactions. Nonetheless, cost-effectiveness and outcomes-based studies could help drive PGx implementation.

## Pharmacogenomics and health disparities: the need for diversity and inclusion

Precision medicine can potentially improve both health outcomes at individual and population levels. Successful implementation strategies to mitigate health disparities warrant careful dissection to ensure equitable and sustainable patient outcomes. While pharmacogenomics may provide a path to close the health gaps across different populations, the same strategy could disadvantage minorities or individuals with a lower scale of socioeconomic status (SES) to garner the benefits of precision medicine (Shaaban and Ji). Indeed, most genome-wide association studies and clinical trials have been conducted in individuals of European ancestry. With limited diversity in genetic and clinical studies, developing informative and inclusive guidelines is challenging. Besides race and ethnicity, sex differences in adverse drug events and the response to therapy are health disparities (Shaaban and Ji). Efforts to increase the representation of women, especially underserved women, could close the sex gap in treatment outcomes or toxicity risk. Disproportionate care access impacts individuals living in certain areas or zip codes, which may compound the disparities in leveraging the benefits of genetic testing or integrating pharmacogenomics into routine patient care.

The state of racial, ethnic, and genetic ancestry diversity in pharmacogenomic studies of smoking cessation also demonstrates the need for diversity and inclusion (Prom-Wormley et al.). To illustrate, most available data are derived from White participants residing in Europe and the United States. Second, four genes were suggested for further investigation, derived from candidate gene association studies, with support from genome-wide association studies. Third, results require additional replication because there was a high degree of variability in reporting and measuring ancestry, race, and ethnicity, and methodological approaches to analyses using these variables (Prom-Wormley et al.). Considerations related to the current landscape of smoking cessation treatment that limit the participation of diverse populations in pharmacogenomic studies need to address the limited access to treatment and the perception of participants and clinicians. Collectively, efforts to diversify the research participants and healthcare settings in pharmacogenomic studies are urgently needed.

## Pharmacogenetic studies in geo-ancestral population subgroups: the Hmong in the United States

Certain chronic diseases show differences in prevalence between populations, and so is the response to treatment. While the reasons behind these differences are multifactorial, population-specific variants may explain some of the drivers in disease prevalence and disparate responses to therapy. The Hmong is an ethnically and demographically distinct Asian subpopulation, underrepresented in genetic or clinical research (Sun et al.). Using a community-based participatory research approach between academia and the community can facilitate community engagement in research to address pressing health needs within the community, such as gout, diabetes, and obesity. Engaging the Hmong community in these research endeavors has uncovered population-specific variants across multiple genes that can influence the response to commonly prescribed medications such as allopurinol, clopidogrel, warfarin, and antidepressants (Sun et al.).

## Pharmacogenomics in rural minnesota: educational workforce training program

Disparities in the uptake of pharmacogenetic testing significantly differ between urban and rural regions. Brown et al. offered a didactic framework to enhance the PGx knowledge base of practicing pharmacists working in rural areas of Minnesota or serving disadvantaged communities. The training program mainly enrolled pharmacists interested in improving health equity, working across different pharmacy specialties that may benefit from PGx implementation, and having no prior PGx education or experience in implementing PGx. After the certificate training program, a large proportion of the enrollees somewhat or strongly agreed that they would apply what they learned in the program to their practice in the following 6 months to a year (Brown et al.). Efforts focused on training pharmacists in rural areas working in underserved communities can become the catalyst for adopting PGx implementation and reducing the access gap in PGx testing between urban and rural settings.

## Pharmacogenetic testing in the medically underserved: the barriers and opportunities

The inverse health equity hypothesis suggests that as new technology, such as PGx testing, is being implemented, it is first accessible to populations with higher SES and delayed access to those with lower SES, who are often in greatest need (Victora et al., 2018). Therefore, assessing the perceptions and attitudes of patients towards pharmacogenetic testing is significant to ensure adequate health communication, facilitate the decision-making process, and improve the patient-physician relationship. Brian et al. sought to assess the feasibility of routine pharmacogenetic testing in medically underserved populations. Insights from such surveys could increase the likelihood of successful PGx implementation. Specifically, the survey was fielded to adults residing in the United States, meeting the poverty guidelines. The survey revealed that most respondents were unfamiliar with PGx testing, but the majority expressed moderate or high interest in receiving one, following an explanation of PGx testing and if to be offered at no cost (Brian et al.). The cost of PGx testing has been a significant limitation for system-wide implementation across different population groups. In the medically underserved, these concerns were even more prevalent. Besides testing costs, there were worries about privacy violations, discrimination in employment, and the possibility of being denied health insurance.

## Future perspectives

Diversity in genetic research will enhance our understanding of heterogeneity in response to therapy and usher in a new paradigm to deliver personalized care. Efforts to increase diversity should engage different perspectives from the community, patients, and providers. Ultimately, increasing diversity and representation in pharmacogenetic research will provide a path for addressing health disparities and realizing the potential of precision health.
